# Amiodarone-Induced Pulmonary Toxicity With Severe Pulmonary Fibrosis Complicated by Stenotrophomonas maltophilia Pneumonia: A Fatal Case Report and Literature Review

**DOI:** 10.7759/cureus.95245

**Published:** 2025-10-23

**Authors:** Sachin Sapkota, Daniel Neri Rosario, Ramesh Acharya, Suchita Acharya, Azucena Del Real, Maria Theresa D. Opina

**Affiliations:** 1 Internal Medicine, Texas Tech University Health Sciences Center El Paso, El Paso, USA; 2 Pulmonology and Critical Care, Texas Tech University Health Sciences Center El Paso, El Paso, USA

**Keywords:** amiodarone, pulmonary fibrosis, pulmonary toxicity, pulse dose steroids, stenotrophomonas maltophilia

## Abstract

Amiodarone is a widely used antiarrhythmic agent, valued for its efficacy and low proarrhythmic risk, but it carries a known potential for serious toxicities, particularly pulmonary toxicity. Amiodarone-induced pulmonary toxicity (APT) remains a life-threatening adverse effect. We report the case of a 75-year-old male with a 30-year history of amiodarone use (200 mg twice daily) who developed progressive respiratory distress consistent with APT. Despite treatment with broad-spectrum antibiotics and corticosteroids, his condition deteriorated. Imaging and clinical findings raised suspicion for APT, which was managed with amiodarone discontinuation and pulse-dose steroid therapy. Subsequently, he developed hospital-acquired *Stenotrophomonas maltophilia* pneumonia, a multidrug-resistant opportunistic infection. Targeted antibiotics were initiated based on sensitivities, but the patient progressed to respiratory failure and multiorgan dysfunction, ultimately resulting in death. This case highlights the diagnostic and therapeutic challenges of managing APT, particularly in elderly patients on long-term therapy. The absence of standardized pulmonary surveillance and delayed recognition can result in advanced disease at presentation. Superimposed nosocomial infections, especially with resistant organisms such as *S. maltophilia*, further complicate outcomes. Clinicians must maintain a high index of suspicion for APT in patients presenting with unexplained respiratory symptoms while on amiodarone. This case underscores the importance of appropriate dosing, consistent outpatient monitoring, and prompt evaluation for opportunistic infections in the setting of clinical deterioration.

## Introduction

Amiodarone is a commonly prescribed antiarrhythmic medication, highly effective for a range of tachyarrhythmias. Despite adverse effects such as thyroid dysfunction, pulmonary toxicity, hepatic toxicity, corneal deposits, optic neuritis, and peripheral neuropathy, its broad antiarrhythmic efficacy, reduction in arrhythmic mortality, and relatively low proarrhythmic risk have contributed to its widespread use [1-5]. Among these adverse effects, amiodarone-induced pulmonary toxicity (APT) is the most serious and lacks a definitive cure [6,7].

Mechanistic studies have demonstrated that APT involves phospholipid accumulation within type II pneumocytes and macrophages, producing injury patterns that range from organizing pneumonia to chronic interstitial fibrosis. The NLRP3 inflammasome pathway has emerged as a central driver of fibrotic signaling via IL-1β and IL-18 cytokine release [8,9]. Experimental evidence also indicates that amiodarone induces significant oxidative stress and mitochondrial injury in lung tissue [10]. Clinically, APT presents with a spectrum of manifestations, from chronic interstitial pneumonitis to acute respiratory distress syndrome (ARDS). Contemporary reports describe severe acute presentations mimicking ARDS, underscoring the critical importance of rapid recognition and immediate drug cessation, as some cases demonstrate dramatic improvement with corticosteroid therapy following amiodarone discontinuation [11,12]. Therefore, a high degree of clinical suspicion is essential, supported by characteristic radiographic findings and the exclusion of other pulmonary diseases [13].

Diagnosing APT can be particularly challenging in hospitalized or critically ill patients, where it may mimic or obscure conditions such as lung infections or preexisting pulmonary disease. Hospital-acquired infections caused by multidrug-resistant organisms are a major concern in intensive care units, given their increasing prevalence and therapeutic challenges [14]. *Stenotrophomonas maltophilia *has emerged as a notable nosocomial respiratory pathogen in critically ill patients, with prolonged hospitalization, mechanical ventilation, corticosteroid use, and invasive airway procedures identified as key risk factors for lower respiratory tract infections in vulnerable populations [15].

We report the case of a 75-year-old male on chronic amiodarone therapy who developed severe pulmonary toxicity, subsequently complicated by hospital-acquired *S. maltophilia *pneumonia. This case highlights the challenges of managing concurrent drug-induced lung injury and opportunistic infection, emphasizing the importance of vigilant outpatient monitoring in patients receiving amiodarone. Furthermore, in severe cases of APT, even pulse-dose steroid therapy may fail to achieve improvement, underscoring the need for future controlled trials to establish standardized treatment guidelines.

## Case presentation

A 75-year-old male with a past medical history of hypertension, coronary artery disease, atrial fibrillation, hyperthyroidism, and diabetes mellitus presented with shortness of breath and a nonproductive cough, ongoing for several months and worsening over the past week. He denied fever, weight loss, appetite loss, or peripheral edema. The patient had a history of chronic amiodarone use (200 mg twice daily for 30 years). He previously worked in copper mines but reported no known exposure to asbestos, silica dust, or household pets.

The patient was initially admitted to an outside hospital, where he received a one-week course of meropenem, linezolid, and methylprednisolone for pneumonia and a suspected COPD exacerbation. He was subsequently transferred to our facility for evaluation of nonresolving pneumonia.

**Figure 1:**
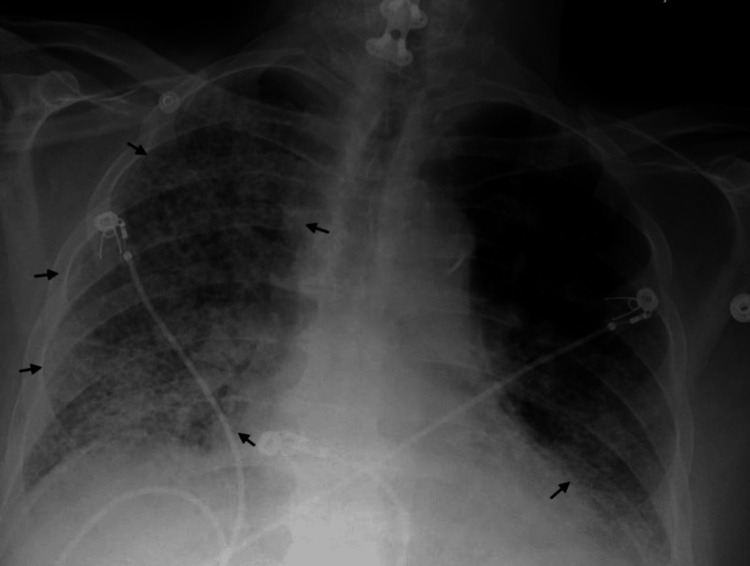
PA view CXR on admission CXR on admission demonstrating a mixed reticular and consolidative pattern throughout both lungs (black arrow), more pronounced in the right hemithorax. CXR, chest X-ray

**Figure 2:**
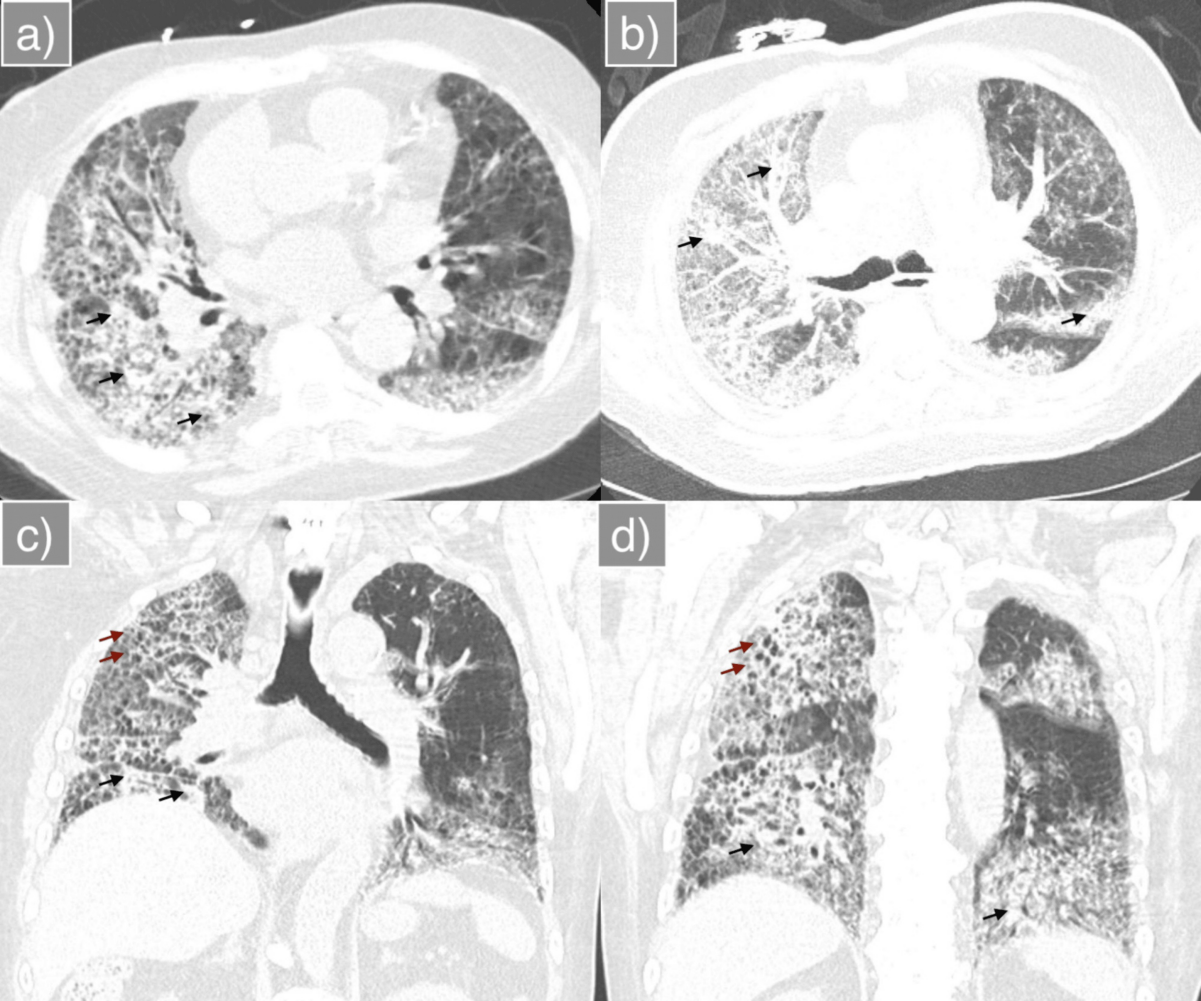
High-resolution CT of the chest demonstrating advanced fibrotic interstitial lung disease (a) Axial section at the mid-lower thorax (atrial level) showing advanced fibrotic changes with diffuse opacities (black arrow) and interstitial thickening, more pronounced on the right. (b) Axial section at the mid-thorax demonstrating similar findings with right-sided predominance. (c) Coronal section at the level of the tracheal bifurcation showing diffuse fibrotic changes, opacities (black arrow), ground-glass opacities, and honeycombing (brown arrow), more prominent on the right. (d) Coronal section anterior to the trachea demonstrating similar advanced fibrotic changes, opacities (black arrow), and honeycombing (brown arrow), predominantly on the right.

**Figure 3:**
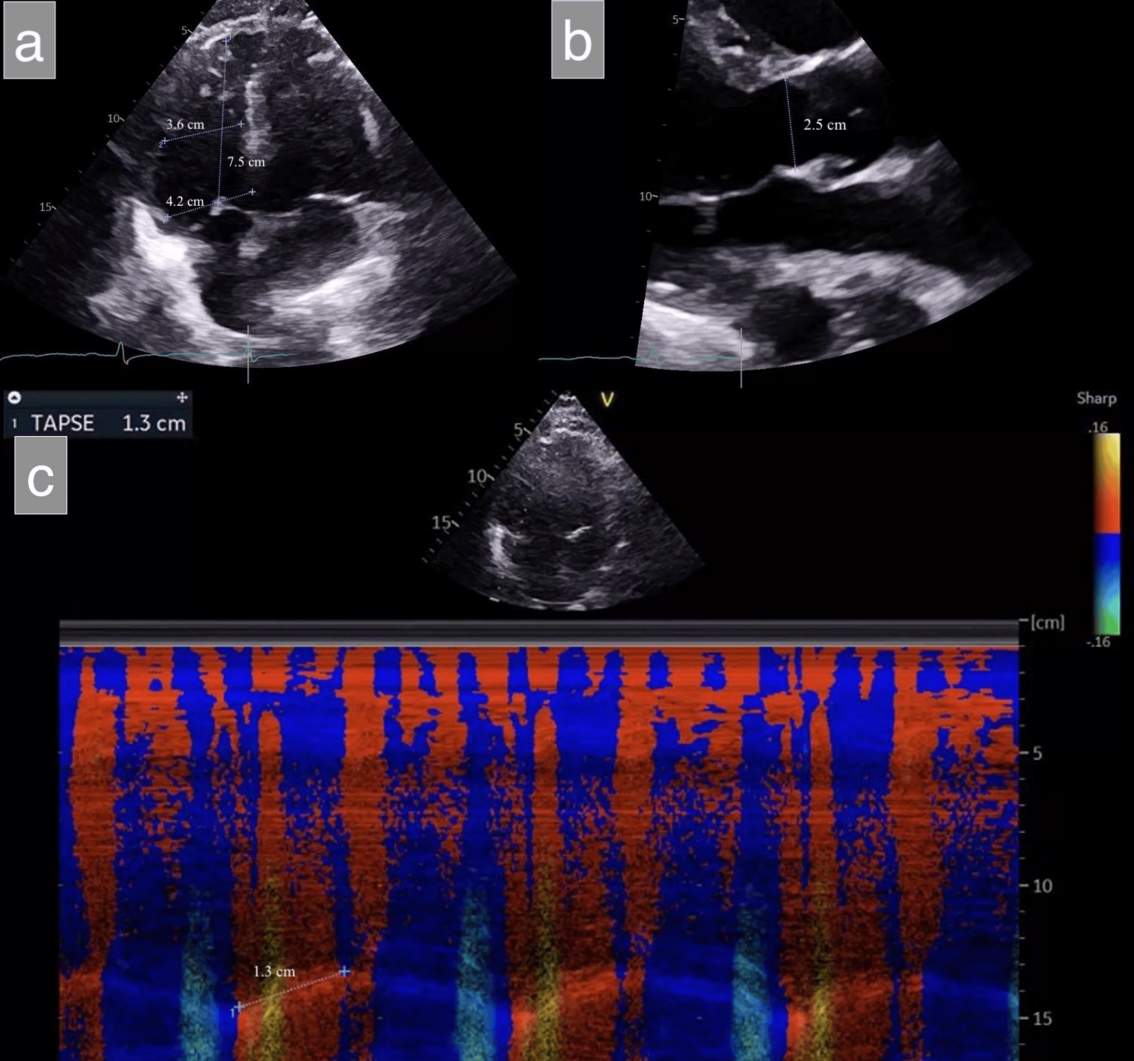
Transthoracic echocardiographic views demonstrating RV dilatation and impaired systolic function with preserved LV function (a) Apical four-chamber view showing a normal LV chamber with a slightly dilated RV. (b) Parasternal long-axis view demonstrating a normal LVOT (diameter 2.5 cm). (c) Color tissue Doppler echocardiogram showing reduced right ventricular systolic function with a TAPSE of 1.3 cm. LV, left ventricle; LVOT, left ventricular outflow tract; RV, right ventricle; TAPSE, tricuspid annular plane systolic excursion

Upon arrival, the patient was in respiratory distress, requiring high-flow nasal cannula (HFNC) at 40 L/min with FiO₂ 90%, but remained hemodynamically stable. Initial laboratory evaluation revealed leukocytosis, elevated erythrocyte sedimentation rate and CRP, and normal procalcitonin (Table 1). Chest X-ray (CXR) demonstrated a mixed reticular and consolidative pattern throughout both lungs (Figure 1). High-resolution CT revealed diffuse bilateral consolidation and interstitial thickening, scattered ground-glass opacities, and honeycombing (Figure 2). CT angiography of the chest, performed at the outside hospital, ruled out pulmonary embolism. Transthoracic echocardiography demonstrated preserved left ventricular systolic function. The right ventricle was dilated with reduced systolic function, and the tricuspid annular plane systolic excursion measured 1.3 cm (Figure 3).

**Table 1:** Laboratory findings on admission and during ICU stay Ab, antibody; Ag, antigen; BUN, blood urea nitrogen; ESR, erythrocyte sedimentation rate; HCO₃, bicarbonate; Hgb, hemoglobin; T4 free, free thyroxine; TSH, thyroid-stimulating hormone

Laboratory test	Reference range	Day 1 (admission)	Day 6	Day 7 (intubated)	Day 11	Day 16 (deceased)
WBC (10³/mm³)	4.5-11	17	24.9	31.9	25.3	21.6
Hgb (g/dL)	12-16	9.7	10.4	9.7	8.2	8.1
HCO₃ (mmol/L)	21-31	35	26	29	26	28
BUN (mg/dL)	6-25	47	35	61	120	93
Creatinine (mg/dL)	0.3-1.3	1.3	0.7	1.8	3.5	2.1
CRP (mg/dL)	0.1-1.0	7.5	6.27	-	10.27	9.45
ESR (mm/hr)	0-9	26	19	33	77	24
Procalcitonin (ng/mL)	≤0.50	0.08	11.97	8.65	1.72	-
T4 free (ng/dL)	0.6-2.0	2.69	1.86	-	1.68	-
TSH (µIU/mL)	0.4-5.0	<0.010	<0.010	-	<0.010	-
Thyroid peroxidase Ab (IU/mL)	0-34	>600	-	-	-	-
Thyroglobulin Ab (IU/mL)	0-0.9	2162.7	-	-	-	-

The patient was evaluated for both infectious and noninfectious causes of unresolved pneumonia. Treatment with meropenem (1 g every eight hours) and linezolid (600 mg every 12 hours) was continued, with the addition of azithromycin (500 mg daily); linezolid was later discontinued after cultures were negative for methicillin-resistant* Staphylococcus aureus*. Given the high clinical suspicion for APT, amiodarone was discontinued, and pulse-dose corticosteroid therapy was initiated with intravenous methylprednisolone 250 mg every six hours for three days, followed by methylprednisolone 60 mg IV daily with a gradual taper. Atrial fibrillation remained well controlled on the patient’s home dose of carvedilol (6.25 mg twice daily), which was later discontinued due to bradycardia and hypotension. Bronchoscopy could not be performed because of the patient’s high oxygen requirements. Extensive infectious and autoimmune workups were negative (Table 2). Despite ongoing therapy, the patient could not be weaned off HFNC, experiencing intermittent desaturation even with minimal activity.

**Table 2:** Comprehensive infectious and autoimmune workup in this case of unresolved pneumonia showing negative findings ANA, antinuclear antibody; c-ANCA, cytoplasmic anti-neutrophil cytoplasmic antibody; CMV, cytomegalovirus; MPO, myeloperoxidase; p-ANCA, perinuclear anti-neutrophil cytoplasmic antibody

Test	Result	Reference range
Influenza and SARS-CoV-2 screen	Negative	Negative
Serum Fungitel beta-D-glucan	<31.25	0.00-60.00
Serum cryptococcal antibody	Negative	<1:20 titer
Serum histoplasma antibody	Negative	<1:1 titer
CMV IgM	Negative	0.00-0.60
CMV IgG	Negative	0.00-30.00
Urine *Streptococcus pneumoniae* antigen	Negative	Negative
Urine *Legionella* antigen	Negative	Negative
*Blastomyces *antibody	Negative	<1:1 titer
Respiratory pathogen panel	Negative	Negative
Serum *Coccidioides *IgM	0.1	<1.0
Serum *Coccidioides *IgG	0.4	<1.0
Serum *Aspergillus* antigen	0.05	0.00-0.49
HIV screen	Negative	Negative
ANA screen	Negative	Negative
p-ANCA	Negative	<1:20 titer
c-ANCA	Negative	<1:20 titer
MPO antibody	<0.2	0.0-0.9
Intrinsic factor blocking antibody	1	0.0-1.1

**Figure 4:**
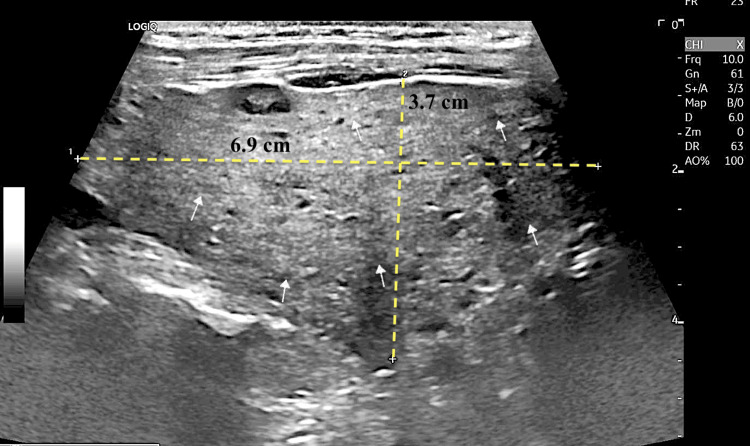
Thyroid ultrasound showing heterogeneous echotexture (white arrows) throughout the right thyroid lobe parenchyma The thyroid is enlarged, with generalized parenchymal heterogeneity, suggestive of diffuse thyroid disease. The right thyroid lobe measures 6.9 × 3.7 cm (yellow dotted lines).

The patient had long-standing hyperthyroidism managed with methimazole (10 mg daily). Thyroid ultrasound demonstrated an enlarged, heterogeneous gland (Figure 4). Thyroid peroxidase and thyroglobulin antibodies were elevated (Table 1), suggesting type I amiodarone-induced thyrotoxicosis. This condition may have significantly worsened respiratory function and overall prognosis, particularly in critically ill or high-risk patients, as observed in our case.

**Figure 5:**
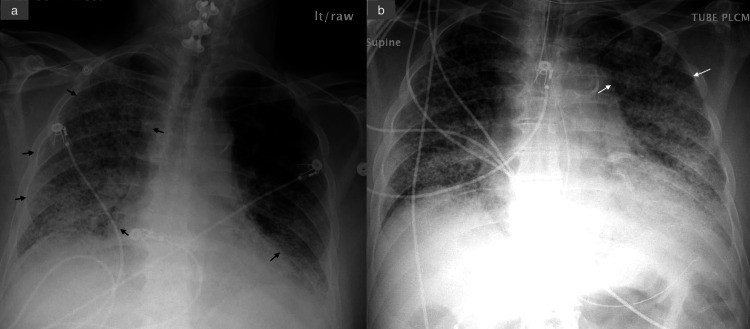
CXR showing progressive worsening of airspace opacities from admission to day 7 (post-intubation) (a) CXR on admission demonstrating a mixed reticular and consolidative pattern throughout both lungs (black arrow), more pronounced in the right hemithorax. (b) Repeat CXR on day 7, after intubation, showing diffusely worsened bilateral pulmonary infiltrates, with significant progression of airspace disease in the left upper lobe (white arrow). CXR, chest X-ray

On hospital day 7, the patient’s pulmonary condition worsened, culminating in a hypoxia-driven cardiac arrest. He was intubated and started on vasopressor support. Subsequently, he developed atrial fibrillation with rapid ventricular response, which was managed with intravenous metoprolol pushes (5 mg as needed), achieving adequate rate control. Follow-up CXR demonstrated worsening bilateral infiltrates (Figure 5). Procalcitonin levels were rising, raising suspicion of opportunistic bacterial infection (Table 1). Respiratory cultures grew *S. maltophilia*, which was not covered by the current antibiotic regimen. Therapy was transitioned to levofloxacin (IV 750 mg every 48 hours, renally dosed), minocycline (oral 100 mg twice daily), and ceftazidime (IV 1000 mg daily), to which the isolate was subsequently confirmed to be susceptible. The antimicrobial susceptibility profile of *S. maltophilia *isolated from respiratory culture is shown in Table 3. First-line therapy with trimethoprim-sulfamethoxazole was deferred due to concerns of renal toxicity.

**Table 3:** Antimicrobial susceptibility profile of Stenotrophomonas maltophilia isolated from respiratory culture The table demonstrates that *S. maltophilia* is susceptible to ceftazidime, levofloxacin, and trimethoprim/sulfamethoxazole. MIC, minimum inhibitory concentration; S, susceptible

Drug	MIC (µg/mL)	Interpretation
Ceftazidime	≤1	S
Levofloxacin	1	S
Trimethoprim/sulfamethoxazole	≤0.5/9.5	S

Despite aggressive interventions, the patient remained in respiratory failure on mechanical ventilation with multiorgan dysfunction. Extracorporeal membrane oxygenation was not available at our institution. On hospital day 15, following a comprehensive goals-of-care discussion with the family, care was transitioned to comfort-focused measures, and the patient ultimately passed away. The family acknowledged that the outcome was inevitable despite all aggressive medical interventions.

## Discussion

Low-dose amiodarone is considered effective for maintaining sinus rhythm but is not used as a first-line agent due to its adverse effects and drug interactions. In patients with heart failure with reduced ejection fraction, amiodarone is preferred, as most other first-line agents, such as flecainide, propafenone, and dronedarone, are contraindicated [1,16]. According to the 2023 AHA/ACC guidelines, APT occurs in 1-2% of patients and is fatal in approximately 10% of cases [1]. A retrospective study reported an incidence of 1.9%, while a large meta-analysis reported 2.9% [17,18]. Although a baseline chest radiograph is recommended prior to initiating amiodarone, no specific surveillance interval for pulmonary toxicity monitoring has been established. Nevertheless, a chest radiograph or CT scan should be obtained if patients develop new or unexplained cough or dyspnea during therapy [1,19]. Pharmacovigilance data from nearly 5,000 reported pulmonary adverse events demonstrate a broad time-to-onset distribution, with median onset ranging from 126 to 227 days, supporting the need for long-term clinical surveillance even after drug cessation due to amiodarone’s prolonged tissue half-life [20].

In our case, initial broad-spectrum antibiotic therapy failed to yield clinical improvement, highlighting the diagnostic and therapeutic challenges of APT. Symptoms are often insidious, and imaging findings overlap with other pulmonary conditions, causing APT to mimic both infectious and noninfectious processes, which can delay diagnosis and management [21]. The pathophysiology of APT involves phospholipid accumulation in type II pneumocytes and macrophages, leading to cellular dysfunction and diverse histologic patterns, including diffuse alveolar damage, interstitial pneumonitis, and organizing pneumonia [8]. Experimental evidence also implicates oxidative stress as a key mediator; animal studies have demonstrated increased lipid peroxidation and decreased antioxidant enzyme activity following amiodarone exposure, with antioxidant interventions showing protective effects against drug-induced alveolar damage [10,22]. Although rare, this adverse reaction can be devastating, as seen in our patient.

Risk factors for APT include cumulative dose, advanced age, and prolonged therapy duration, all present in our case [17,23]. A daily dose of 200 mg is typically adequate for most patients but may require individualized adjustment [24]. The absence of a well-defined safety margin and poor correlation between serum levels and toxicity contribute to delayed recognition of pulmonary toxicity. Diagnosis is usually based on clinical suspicion, imaging features, and exclusion of other pulmonary causes. Histological confirmation through biopsy can be obtained, but it is often high-risk in hypoxic patients, as was the case here. Bronchoscopy with bronchoalveolar lavage is commonly performed to exclude infection and may reveal foamy macrophages, although these findings are supportive rather than diagnostic of APT [11,25,26]. Pulmonary function tests typically show a restrictive pattern with reduced diffusion capacity [13,24,27].

Following diagnosis, management of APT involves amiodarone discontinuation and corticosteroids (initial dosing of 0.75-1.0 mg/kg/day of oral prednisolone) tapered over several weeks to months [1,21,28]. Although controlled trial data are lacking, case reports consistently document clinical and radiographic improvement with high-dose corticosteroid therapy [11,29-31]. In some instances of rapidly progressive disease, pulse-dose therapy with methylprednisolone (1,000 mg/day) has been reported to be effective [12,32-34].

Despite appropriate discontinuation of amiodarone and corticosteroid therapy, our patient acutely decompensated due to superimposed multidrug-resistant *S. maltophilia*. *S. maltophilia *is an emerging nosocomial pathogen with increasing prevalence and antimicrobial resistance. Although often considered less virulent than other hospital-acquired bacteria, it can be devastating in high-risk patients, particularly those with underlying pulmonary disease, prolonged hospitalization, mechanical ventilation, or prior broad-spectrum antibiotic use, all of which were present in our patient [35-37]. Recent retrospective studies report mortality rates of 13.7% among all hospitalized patients and up to 49.7% in critically ill ICU patients [38,39]. In immunocompromised individuals, especially those with hematologic malignancies, *S. maltophilia *can cause fulminant hemorrhagic pneumonia with rapid progression and exceptionally high case-fatality rates [40].

In our case, initial antibiotic therapy broadly covered most pathogens but did not target *S. maltophilia*, an organism intrinsically resistant to many antibiotics due to mechanisms such as beta-lactamase production and efflux pumps [35]. The 2024 Clinical and Laboratory Standards Institute (34th edition) reports established breakpoints for six agents against *S. maltophilia*: cefiderocol, chloramphenicol, levofloxacin, minocycline, ticarcillin-clavulanate, and trimethoprim-sulfamethoxazole [35]. Recent multicenter studies suggest that while monotherapy with trimethoprim-sulfamethoxazole remains effective in many cases, combination therapy may provide a survival benefit in immunocompromised patients and those with high illness severity scores [41]. Although APT and *S. maltophilia *infection are distinct processes, underlying lung injury from APT and high-dose steroid therapy may predispose to opportunistic infection.

## Conclusions

This fatal case underscores the critical importance of ensuring appropriate amiodarone dosing, with ongoing efforts to identify the lowest effective maintenance dose. Structured follow-up is essential for early detection of APT. Although amiodarone remains widely used, standardized monitoring and surveillance protocols are imperative. Corticosteroids are frequently employed, and case reports suggest benefit, but the absence of randomized controlled trials limits the development of standardized treatment protocols. In severe APT, even pulse-dose steroids may fail to achieve improvement, highlighting the urgent need for well-designed trials to guide evidence-based management. Additionally, this case emphasizes the importance of considering superimposed infections in patients with suspected APT who deteriorate despite appropriate initial management. Early recognition and targeted antimicrobial therapy, based on local resistance patterns and culture data, are crucial; however, outcomes may still be poor in severe cases.

## References

[1] Joglar JA, Chung MK, Armbruster AL (2024). 2023 ACC/AHA/ACCP/HRS guideline for the diagnosis and management of atrial fibrillation: a report of the American College of Cardiology/American Heart Association Joint Committee on Clinical Practice Guidelines. Circulation.

[2] Hohnloser SH, Kuck KH, Lilienthal J (2000). Rhythm or rate control in atrial fibrillation—pharmacological intervention in atrial fibrillation (PIAF): a randomised trial. Lancet.

[3] Strickberger SA, Hummel JD, Bartlett TG (2003). Amiodarone versus implantable cardioverter-defibrillator:randomized trial in patients with nonischemicdilated cardiomyopathy and asymptomaticnonsustained ventricular tachycardia—AMIOVIRT. J Am Coll Cardiol.

[4] Mujović N, Dobrev D, Marinković M, Russo V, Potpara TS (2020). The role of amiodarone in contemporary management of complex cardiac arrhythmias. Pharmacol Res.

[5] Singh BN (1996). Antiarrhythmic actions of amiodarone: a profile of a paradoxical agent. Am J Cardiol.

[6] Martin WJ 2nd, Rosenow EC 3rd (1988). Amiodarone pulmonary toxicity. Recognition and pathogenesis (Part 2). Chest.

[7] Șorodoc V, Indrei L, Dobroghii C (2024). Amiodarone therapy: updated practical insights. J Clin Med.

[8] Budin CE, Cocuz IG, Sabău AH, Niculescu R, Ianosi IR, Ioan V, Cotoi OS (2022). Pulmonary fibrosis related to amiodarone—is it a standard pathophysiological pattern? A case-based literature review. Diagnostics (Basel).

[9] Colunga Biancatelli RM, Solopov PA, Catravas JD (2022). The inflammasome NLR family pyrin domain-containing protein 3 (NLRP3) as a novel therapeutic target for idiopathic pulmonary fibrosis. Am J Pathol.

[10] Dawood SA, Asseri AA, Shati AA, Eid RA, El-Gamal B, Zaki MS (2024). L-carnitine ameliorates amiodarone-mediated alveolar damage: oxidative stress parameters, inflammatory markers, histological and ultrastructural insights. Pharmaceuticals (Basel).

[11] Rodriguez-Rivera AA, Chavez Melendez DA, Sultan S, Goyal A, Sharma B (2023). Amiodarone-induced pulmonary toxicity leading to acute respiratory distress syndrome. Chest.

[12] Nacca N, Bhamidipati CM, Yuhico LS, Pinnamaneni S, Szombathy T (2012). Severe amiodarone induced pulmonary toxicity. J Thorac Dis.

[13] Wolkove N, Baltzan M (2009). Amiodarone pulmonary toxicity. Can Respir J.

[14] Teng J, Imani S, Zhou A (2023). Combatting resistance: understanding multi-drug resistant pathogens in intensive care units. Biomed Pharmacother.

[15] Wang Y, Wang Y, Rong H, Guo Z, Xu J, Huang X (2022). Risk factors of lower respiratory tract infection caused by Stenotrophomonas maltophilia: systematic review and meta-analysis. Front Public Health.

[16] Deedwania PC, Singh BN, Ellenbogen K, Fisher S, Fletcher R, Singh SN (1998). Spontaneous conversion and maintenance of sinus rhythm by amiodarone in patients with heart failure and atrial fibrillation: observations from the Veterans Affairs Congestive Heart Failure Survival Trial of Antiarrhythmic Therapy (CHF-STAT). Circulation.

[17] Kwok WC, Ma TF, Chan JW, Pang HH, Ho JC (2022). A multicenter retrospective cohort study on predicting the risk for amiodarone pulmonary toxicity. BMC Pulm Med.

[18] Piccini JP, Berger JS, O'Connor CM (2009). Amiodarone for the prevention of sudden cardiac death: a meta-analysis of randomized controlled trials. Eur Heart J.

[19] Goldschlager N, Epstein AE, Naccarelli GV, Olshansky B, Singh B, Collard HR, Murphy E (2007). A practical guide for clinicians who treat patients with amiodarone: 2007. Heart Rhythm.

[20] Yang J, Zhang G, You M (2024). Pulmonary adverse events associated with amiodarone: a real-world pharmacovigilance study based on the FDA adverse event reporting system. Expert Opin Drug Saf.

[21] Schwaiblmair M, Berghaus T, Haeckel T, Wagner T, von Scheidt W (2010). Amiodarone-induced pulmonary toxicity: an under-recognized and severe adverse effect?. Clin Res Cardiol.

[22] Ahmed D, Youssef MY, Emam NM (2020). Oxidative stress in amiodarone-induced pulmonary toxicity in rats and the protective effect of L-carnitine and vitamin C. Mansoura J Forensic Med Clin Toxicol.

[23] Papiris SA, Triantafillidou C, Kolilekas L, Markoulaki D, Manali ED (2010). Amiodarone: review of pulmonary effects and toxicity. Drug Saf.

[24] van Erven L, Schalij MJ (2010). Amiodarone: an effective antiarrhythmic drug with unusual side effects. Heart.

[25] Bedrossian CW, Warren CJ, Ohar J, Bhan R (1997). Amiodarone pulmonary toxicity: cytopathology, ultrastructure, and immunocytochemistry. Ann Diagn Pathol.

[26] Adams PC, Gibson GJ, Morley AR, Wright AJ, Corris PA, Reid DS, Campbell RW (1986). Amiodarone pulmonary toxicity: clinical and subclinical features. Q J Med.

[27] Pitcher WD (1992). Amiodarone pulmonary toxicity. Am J Med Sci.

[28] Epstein AE, Olshansky B, Naccarelli GV, Kennedy JI Jr, Murphy EJ, Goldschlager N (2016). Practical management guide for clinicians who treat patients with amiodarone. Am J Med.

[29] Garg J, Agrawal N, Marballi A, Agrawal S, Rawat N, Sule S, Lehrman SG (2012). Amiodarone induced pulmonary toxicity: an unusual response to steroids. Am J Case Rep.

[30] Fabiani I, Tacconi D, Grotti S, Brandini R, Salvadori C, Caremani M, Bolognese L (2011). Amiodarone-induced pulmonary toxicity mimicking acute pulmonary edema. J Cardiovasc Med (Hagerstown).

[31] Abuzaid A, Saad M, Ayan M, Kabach A, Haddad TM, Smer A, Arouni A (2015). Acute amiodarone pulmonary toxicity after drug holiday: a case report and review of the literature. Case Rep Cardiol.

[32] Umetani K, Abe M, Kawabata K, Iida T, Kohno I, Sawanobori T, Kugiyama K (2002). SP-D as a marker of amiodarone-induced pulmonary toxicity. Intern Med.

[33] Covi S, Clark J, Delius R, Chauhan M (2019). Amiodarone toxicity in two post-operative congenital heart disease patients. Prog Pedia Cardiol.

[34] Tan C, Kumar P (2023). Too little, too late: a case of a swift fatal culmination of amiodarone induced pulmonary toxicity in an adult male. Int Med Case Rep J.

[35] Tamma PD, Heil EL, Justo JA, Mathers AJ, Satlin MJ, Bonomo RA (2024). Infectious Diseases Society of America 2024 guidance on the treatment of antimicrobial-resistant gram-negative infections. Clin Infect Dis.

[36] Huang C, Lin L, Kuo S (2024). Risk factors for mortality in Stenotrophomonas maltophilia bacteremia - a meta-analysis. Infect Dis (Lond).

[37] Nseir S, Di Pompeo C, Brisson H (2006). Intensive care unit-acquired Stenotrophomonas maltophilia: incidence, risk factors, and outcome. Crit Care.

[38] Appaneal HJ, Lopes VV, LaPlante KL, Caffrey AR (2023). Treatment, clinical outcomes, and predictors of mortality among a national cohort of hospitalized patients with Stenotrophomonas maltophilia infection. Public Health.

[39] Guerci P, Bellut H, Mokhtari M (2019). Outcomes of Stenotrophomonas maltophilia hospital-acquired pneumonia in intensive care unit: a nationwide retrospective study. Crit Care.

[40] Huang C, Kuo S, Lin L (2024). Hemorrhagic pneumonia caused by Stenotrophomonas maltophilia in patients with hematologic malignancies—a systematic review and meta-analysis. Medicina (Kaunas).

[41] Chen L, Hua J, Hong S (2023). Assessment of the relative benefits of monotherapy and combination therapy approaches to the treatment of hospital-acquired Stenotrophomonas maltophilia pneumonia: a multicenter, observational, real-world study. Ann Intensive Care.

